# Increased cerebrospinal fluid soluble TREM2 concentration in Alzheimer’s disease

**DOI:** 10.1186/s13024-016-0071-x

**Published:** 2016-01-12

**Authors:** Amanda Heslegrave, Wendy Heywood, Ross Paterson, Nadia Magdalinou, Johan Svensson, Per Johansson, Annika Öhrfelt, Kaj Blennow, John Hardy, Jonathan Schott, Kevin Mills, Henrik Zetterberg

**Affiliations:** Department of Molecular Neuroscience, UCL Institute of Neurology, Queen Square, London, WC1N 3BG UK; UCL Institute of Child Health, Guilford Street, London, WC1N 1EH UK; Dementia Research Centre UCL Institute of Neurology, Queen Square, London, WC1N 3BG UK; Department of Internal Medicine, Sahlgrenska Academy, University of Gothenburg, Gothenburg, S-413 45 Sweden; Department of Endocrinology, Skaraborg Hospital, Skövde, S-541 85 Sweden; Department of Psychiatry and Neurochemistry, Institute of Neuroscience and Physiology, the Sahlgrenska Academy at the University of Gothenburg, Mölndal, S-431 80 Sweden

**Keywords:** Alzheimer’s disease, Microglia, Cerebrospinal fluid, TREM2

## Abstract

**Background:**

The discovery that heterozygous missense mutations in the gene encoding triggering receptor expressed on myeloid cells 2 (TREM2) are risk factors for Alzheimer’s disease (AD), with only the apolipoprotein E (APOE) ε4 gene allele conferring a higher risk, has led to increased interest in immune biology in the brain. TREM2 is expressed on microglia, the resident immune cells of the brain and has been linked to phagocytotic clearance of amyloid β (Aβ) plaques. Soluble TREM2 (sTREM2) has previously been measured in cerebrospinal fluid (CSF) by ELISA but in our hands commercial kits have proved unreliable, suggesting that other methods may be required. We developed a mass spectrometry method using selected reaction monitoring for the presence of a TREM2 peptide, which can be used to quantify levels of sTREM2 in CSF.

**Findings:**

We examined CSF samples from memory clinics in Sweden and the UK. For all samples the following were available: clinical diagnosis, age, sex, and measurements of the CSF AD biomarkers Aβ42, T-tau and P-tau_181_. AD patients (*n* = 37) all met biomarker (IWG2) criteria for AD. Control individuals (*n* = 22) were cognitively normal without evidence for AD in CSF. We found significantly higher sTREM2 concentration in AD compared to control CSF. There were significant correlations between CSF sTREM2 and T-tau as well as P-tau_181_. CSF sTREM2 increase in AD was replicated in a second, independent cohort consisting of 24 AD patients and 16 healthy volunteers.

**Conclusion:**

CSF concentrations of sTREM2 are higher in AD than in controls, and correlate with markers of neurodegeneration. CSF sTREM2 may be used to quantify glial activation in AD.

**Electronic supplementary material:**

The online version of this article (doi:10.1186/s13024-016-0071-x) contains supplementary material, which is available to authorized users.

## Introduction

Homozygous mutations in the triggering receptor expressed on myeloid cells 2 (TREM2) are known to cause polycystic lipomembranous osteodysplasia with sclerosing leukoencephalopathy, also known as Nasu-Hakola disease (NHD) [1, 2]. The disease is initially characterised by ankle swellings and frequent bone fractures, however as the disease progresses a neurological syndrome develops that resembles the behavioural variant of frontotemporal dementia (bFTD) [3]. Recently, dementia-like symptoms without bone involvement have been reported in patients with homozygous missense mutations in the *TREM2* gene [4]. Further to this, heterozygous mutations in the *TREM2* gene have been identified as risk factors for Alzheimer’s disease (AD), Parkinson’s disease (PD), amyotrophic lateral sclerosis (ALS) and FTD [5–9], although a recent study utilizing detailed clinical phenotyping and *TREM2* sequencing found that the most disease-associated mutation (R47H) is a risk factor only for AD and not other neurodegenerative diseases [10]. However, these findings still suggest a crucial role for TREM2 in maintaining homeostasis and modulating inflammation in the brain.

TREM2 is a receptor glycoprotein of 230 amino acids which belongs to the immunoglobulin superfamily. In the brain, TREM2 is expressed exclusively by myeloid cells which include microglia, the brain’s resident immune cells [11]. *In vitro*, TREM2 promotes phagocytosis, suppresses toll like receptor-induced inflammatory cytokine production and enhances anti-inflammatory cytokine transcription [12, 13].

Given the role TREM2 plays in immune function and the increased risk of disease that variants cause, we set out to investigate TREM2 levels in the CSF of well characterised AD cases and compare them to controls using a novel selected reaction monitoring (SRM) technique.

## Methods

### Subjects

The study included 37 AD patients who fulfilled the revised proposed International Working Group (IWG2) criteria [14] in which, for research purposes, a clinical diagnosis of AD should be supported by a typical AD biomarker profile of concomitant tau and Aβ pathology. Neurodegeneration and neurofibrillary tangle pathology are reflected in the CSF by increased total tau (T-tau) and phospho-tau (P-tau) concentrations, respectively [15–18] whereas cerebral Aβ plaque pathology is reflected by reduced CSF levels of the 42 amino acid-long, aggregation-prone Aβ protein (Aβ42) [19, 20]. To determine AD pathology, we used the cut-offs for CSF T-tau, P-tau and Aβ42 established by Duits et al., *i.e.*, a CSF total tau/Αβ42 ratio of more than 0.52, and an Aβ42 concentration of less than 550 pg/ml [21]. Further, 22 control individuals who were cognitively normal and had a negative CSF AD biomarker profile were included. Controls from Sweden were all cognitively normal volunteers (*n* = 16). The remaining controls from the UK cohort were patients who sought treatment for non-neurodegenerative disorders including depression. One of the AD patients was considered an outlier due to excessively high T-tau and P-tau measurements (T-tau 5894 pg/ml, P-tau 363 pg/ml) and was removed from the analysis. This did not affect the results. In the same round of experiments as for the original AD-control analyses, 6 patients with other neurodegenerative diseases were analysed (2 patients with FTD, 2 patients with semantic dementia, 1 patient with corticobasal syndrome (CBS) and 1 patient with dementia with Lewy bodies (DLB). These were diagnosed according to standard clinical criteria as previously described [22, 23]. Twenty-four AD patients and 16 healthy volunteers, diagnosed according to the same criteria as for the discovery cohort, were used to test for replication of the findings. Neither patients nor controls were on cortisol treatment, but there was no information on the potential use of non-steroidal anti-inflammatory drugs.

The study was approved by the regional ethics committees at UCL and the University of Gothenburg.

### Experimental protocols

CSF was collected prospectively in polypropylene tubes according to standard operating procedures, in the morning, at the two sites, then aliquoted into polypropylene tubes and frozen within 2 hours of being collected. After being stored at -80 °C CSF was thawed, then 100ul was pipetted off, freeze dried, and subjected to in-solution digestion. Twenty μl of digest buffer was added (100 mM Tris, pH 7.8 containing 6 M urea, 2 M thiourea and 2 % amidosulfobetaine-14) plus yeast enolase as an internal standard. Protein disulphide bridges were reduced by the addition of 1.5 μL of 100 mM Tris-HCl pH 7.8 containing 5 M dithiothreitol and free thiol groups were carboamidomethylated by incubation with 3 μL of 100 mM Tris-HCl pH 7.8 containing 5 M iodoacetamide. The solution was diluted to a final volume of 200 μl, vortexed and 1 μg of sequence grade trypsin was added. Samples were then incubated for 12-16 hr in a water bath at 37 °C.

**Fig. 1:**
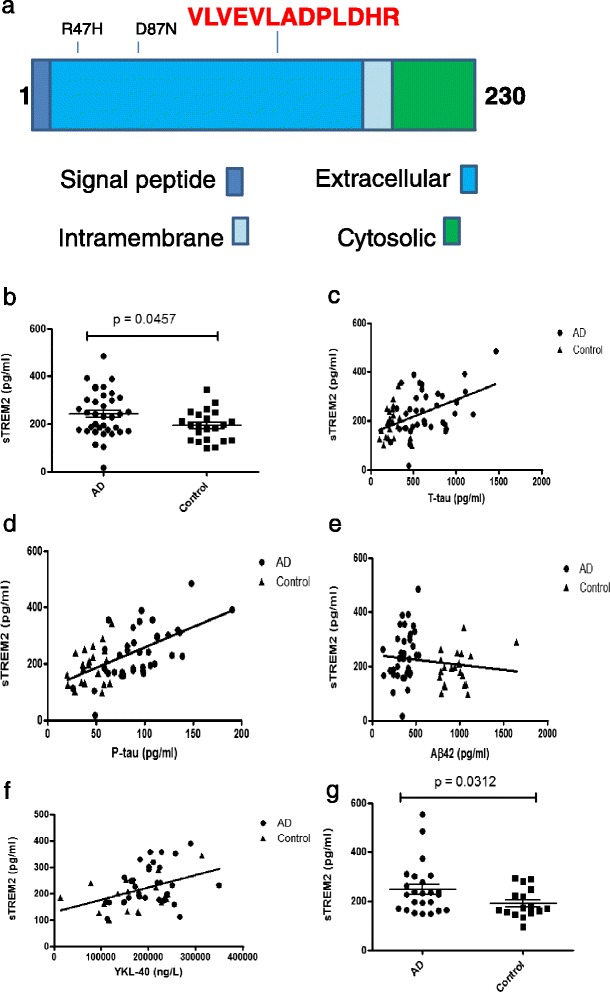
Increased CSF sTREM2 concentration in AD. **a** shows a cartoon of the TREM2 peptide indicating in red the peptide sequence and location. **b** SRM-based analysis of sTREM2 levels in AD cases and controls shows sTREM2 present in all samples (*n* = 59) but a significant increase (*p* = 0.0457) is observed in AD cases (*n* = 37). **c** There is a significant correlation between CSF T-tau and sTREM2 (r = 0.3863 *p* = 0.0023). This is also seen (**d**) when comparing P-tau and sTREM2 (r = 0.5331 *p* = < 0.0001). However, there is no significant correlation between sTREM2 levels and Aβ42 (r = -0.2097 *p* = 0.1280) (**e**). Levels of YKL-40 correlate positively with sTREM2 levels (f) (r = 0.3271 *p* = 0.0204). **g** In a second cohort we replicate the finding that CSF sTREM2 levels are increased in AD cases (*p* = 0.0312)

In order to design a single reaction monitoring (SRM) assay for TREM2, a unique 13 amino acid-long TREM2 peptide was synthesized. This peptide was chosen so that it was not subject to post-translational modifications such as phosphorylation or glycosylation and not affected by known genetic polymorphisms or mutations (Fig. 1a). The assay was established on a Waters Acquity UPLC coupled to a Xevo TQ-S triple quadrupole mass spectrometer, as previously described [24]. The instrument was operated in positive ion mode. The capillary voltage was maintained at 3.7 kV, with source temperature held constant at 150 °C and nitrogen used as the nebulising gas at a flow rate of 30 L/h. For the TREM2 peptide, precursor ion masses were determined in scan mode with product ion masses determined following collision-induced dissociation with argon. Two transitions were selected for confirmatory purposes; clean transitions without interfering peaks were used. Custom synthesised peptides (Genscript, USA) were used to optimise the peptide detection and to determine the retention time and identify unequivocally the correct peaks/s in CSF. A single 10ul volume of each CSF digest was injected onto a Waters CORTECS UPLC C18 + Column, 90 Å, 3 mm × 100 mm column attached to a C18+ VanGuard pre-column. UPLC and MS tune conditions were performed as described previously [25]. Dynamic Multiple Reaction Monitoring was performed over a 10 minute gradient with a minimum of 0.01 sec dwell time for quantitative transitions and a minimum of 12 data points per peak on Waters Xevo TQ-S MS.

QC runs of pooled CSF digests were run in triplicate at the start of the run and then every 10 injections. A CV within 10 % for each QC was considered acceptable. CSF was spiked with sTREM2 peptides to create a calibration curve with average concentrations of biomarker levels and analysed for intra- and inter-batch variations that were below 10 %. The curve was linear up to 40 pmols.

CSF T-tau, P-tau and Aβ42 were analysed using INNOTEST enzyme-linked immunosorbent assays (ELISAs) (Fujirebio Europe N.V., Gent, Belgium). Plasma C-reactive protein (CRP) was measured using a commercial immunoturbidimetry assay on a Cobas 6000 instrument according to the manufacturer’s instructions (Roche Diagnostics, Penzberg, Germany). CSF YKL-40 and CCL2 were measured with commercially available enzyme-linked immunosorbent assays (R&D Systems, Minneapolis, Minnesota, USA) according to the manufacturer’s instructions.

### Data and statistical analysis

Mass spectrometry data were analysed using Mass Lynx version 4.1 and GraphPad Prism. D’Agostino-Pearson test for normality was performed on all data. As most of the parameters displayed skewed distributions, non-parametric tests were used for all comparisons (Mann Whitney U) and correlations (Spearman’s rank). Gender differences were assessed by χ^2^ test. Threshold for significance was set at *p* < 0.05.

## Results

There were no significant differences in gender, age or plasma CRP breakdown between AD and control groups (Table 1). Despite there being a trend towards higher plasma CRP levels in controls, there was no correlation between plasma CRP levels and CSF sTREM2 levels in AD patients or controls (r = -0.004833 *p* = 0.9764).

**Table 1:** Characteristics of AD patients and controls

Subject details	Controls (*n* = 22)	AD patients (*n* = 37)	*p* value
Gender (F/M), no (%)	10(45)/12(55)	19(53)/18(47)	0.6
Age, years (mean ± SD)	69.2 ± 8.0	70.51 ± 7.5	0.54
MMSE score (median IQR)	29.00(26.50-29.00)	22.00(18.00-25.00	<0.0001
*APOE* ε4 positive (%)	33 %	67 %	0.0002
Plasma CRP (mg/L), median (IQR)	2.095 (0.8425 – 4.7535) *n* = 16	1.020 (0.6500 – 1.295) n = 25	0.0543
CSF Biomarkers			
Aβ1-42 (pg/ml), median (IQR)	978.5(821.8-1045)	367.0(301.0-433.0)	<0.0001
T-tau (pg/ml), median (IQR)	239.5(196.3-279.0)	596.0(455.0-858.5)	<0.0001
P-tau_181_ (pg/ml), median (IQR)	45.0(36.8-58.3)	95.0(75.0-111.5)	<0.0001
T-tau/Aβ1-42 ratio, median (IQR)	0.25(0.19-0.33)	1.56(1.24-2.55)	<0.0001
sTREM2 (pg/ml), median (IQR)	195.6(131.0-240.7)	231.2(172.5-305.4)	0.0457

Data expressed as mean ± SD or median (IQR) as appropriate. Probability values (p) denote differences between control and AD. A χ^2^ test was used for gender and *APOE* genotype comparisons. CSF biomarkers and sTREM2 were evaluated using the Mann-Whitney U test

As expected, the AD subjects had significantly lower MMSE scores and Aβ42 levels, and significantly higher concentrations of T-tau, P-tau and T-tau/Aβ42 ratios compared with controls (Table 1). CSF sTREM2 concentrations were significantly higher in AD patients compared with controls (Table 1, Fig. 1b, *p* = 0.0457). Analysing the AD and control samples separately, P-tau was positively correlated with sTREM2 in the AD subjects (*p* = 0.0002) but not in controls. There were no significant correlations between sTREM2 and either T-tau or Aβ42; there was also no correlation between MMSE score and sTREM2 levels in either AD patients or controls. However, when analyzing AD and control individuals together, there was a positive correlation of sTREM2 levels with both T-tau (Fig. 1c, r = 0.3863 *p* = 0.0023) P-tau (Fig. 1d, r = 0.5331 *p* = < 0.0001) but not with Aβ42 (Fig. 1e). Cardiovascular risk factors such as stroke, hypertension and smoking were not associated with CSF sTREM2 concentration (*p* > 0.05 for all comparisons). In a subset (52 subjects), CSF sTREM2 correlated positively with YKL-40 (r =0.3271, *p* = 0.0204, Fig. 1f) and a similar trend was seen for CSF CCL2, although not significant (r = 0.28, *p* = 0.07). CSF sTREM2 concentrations were similar in *APOE* ε4-positive and *APOE* ε4-negative AD patients (*p* = 0.5917), as well as in *APOE* ε4-positive and *APOE* ε4-negative control individuals (*p* = 0.2735). In the same round of analyses, CSF sTREM2 was measured in 6 patients with other neurodegenerative diseases (2 patients with FTD, 2 patients with semantic dementia, 1 patient with CBS and 1 patient with DLB). The one CBS case was the only patient with a sTREM2 concentration above 200 pg/mL. The other patients all had control-like concentrations (Additional file 1: Table S1). We replicated the results in an independent study of 24 patients with AD and 16 healthy controls, again showing elevated CSF sTREM2 in AD (Fig. 1 g, *p* = 0.0312 and Additional file 2: Table S2).

## Discussion

Since the publication of papers attributing a higher risk of AD in those carrying variants in the *TREM2* gene [5, 26], an enormous amount of work has focused on this gene and its protein product. *TREM2* variants have also been implicated in the risk for other neurodegenerative diseases such as PD [6, 7], FTD [7, 8] and ALS [9]. These findings implicate TREM2 in the pathogenesis of AD and likely other neurodegenerative diseases. Here, we show that CSF concentrations of sTREM2 are increased in AD and correlate with markers of neurodegeneration and glial activation, but not amyloid deposition.

In their recent paper, Kleinberger et al. [27] used an in-house ELISA to measure levels of sTREM2 in patients with AD and FTD, as well as in patients with homozygous *TREM2* mutations that cause or are predicted to cause loss of function of TREM2 (p.T66M and p.Q33X). Whilst it was impossible to measure sTREM2 in the CSF or serum of the homozygous mutation patients, they did find reduction in CSF sTREM2 in the AD patients [27]. We found these results surprising for a number of reasons. First, *TREM2* variants associated with AD and reduced TREM2 secretion are rare and should not influence CSF levels in a downward manner in sporadic AD. Second, a study on a number of different mouse models of AD, which measured mRNA expression in different parts of the brain, showed expression of the *Trem2* gene to be increased in cortex, cerebellum, and hippocampus in all models by 18 months of age [28]. Finally, as inflammation has been linked to AD in many studies (for review see [29]), one would expect that CSF markers of neuroinflammation – and so perhaps CSF sTREM2 concentration – would be increased rather than decreased in AD. This is supported by the finding that CSF concentrations of sTREM2 in patients with multiple sclerosis (MS) and other inflammatory diseases were found to be higher than concentrations measured in CSF from non-inflammatory neurologic disease patients [30], corroborating that the marker reflects neuroinflammatory processes in the CNS. Prior to using the method we developed for this study, we attempted to use a commercial ELISA to measure sTREM2 but were unable to validate it.

To explore the relationship between CSF sTREM2 and AD in more detail, we aimed to combine detailed phenotyping of patients and controls with highly accurate measurement of sTREM2. For the former we included conventional CSF biomarkers to ensure as far as possible that individuals with AD had AD pathology; and conversely to ensure a pure control group who did not have evidence of asymptomatic AD pathology. For the latter, we used a very sensitive recently developed SRM assay [24] and adapted it to measure sTREM2. Using these methods, we found a significant increase in sTREM2 concentrations in the CSF of AD subjects and, additionally, we showed that sTREM2 concentrations correlated positively with T-tau and P-tau concentrations in the whole data set, whereas no such correlation was seen for CSF Aβ42. Whilst the correlation was lost for T-tau when considering AD patients separately, the correlation between sTREM2 and P-tau remained. Further, we showed that sTREM2 does not seem to depend on *APOE* genotype, as there was no difference in CSF sTREM2 concentrations between *APOE* ε4 carriers and non-carriers. Finally, a positive correlation of CSF sTREM2 with the astroglial marker CSF YKL-40 [31, 32] and a trend for a similar correlation with CSF CCL2, a putative microglial marker [33], support that CSF sTREM2 may reflect glial activation. It may be argued that YKL-40 is expressed in both astrocytes and microglia [31, 32] and that it therefore is hard to dissect the individual contribution of astrocytes and microglia to CSF sTREM2 elevation. However, so far TREM2 expression has only been observed in microglia and not in astrocytes or other cell types of the brain [34, 35]. We therefore find it likely that most CSF sTREM2 represents release from microglia, although we cannot rule out interplay between activated astrocytes and microglia in the release mechanism. In regards to disease specificity, our data prevent us from making a conclusive statement, but it is interesting to note that among the 6 cases with neurodegenerative diseases other than AD, only the one CBS patient (a condition that often displays AD pathology) had a high CSF sTREM2 concentration, whereas the other 5 cases had CSF sTREM2 concentrations in the lower control range. Cases for these other neurodegenerative diseases will be collected so a properly powered study can be conducted in the future.

There are many potential roles for inflammation in the pathogenesis of AD with one possibility being that Aβ deposition occurs independent of inflammatory processes, but that the type and extent of inflammatory response to Aβ deposition within the brain might trigger or influence subsequent neurodegeneration [36]. Our data provide support for this hypothesis: the absence of correlation of CSF sTREM2 and Aβ42 suggests that increased sTREM2 release from microglia is not primarily related to Aβ deposition; whilst the relationship between CSF sTREM2 and tau proteins in particular suggests that an increased inflammatory response is related to neurodegeneration. The mechanism for the release of sTREM2, and also T-tau and P-tau, to the CSF is not known in detail. CSF T-tau concentrations probably reflect the intensity of neuronal damage and degeneration, with higher levels correlating with amount of damaged tissue, rate of progression and mortality [37], *i.e.* higher amounts of tau are released to CSF with more intense neurodegeneration. For CSF P-tau, the situation is more unclear, since some studies report correlations between high P-tau levels and cortical tangle pathology, while others studies, similar to T-tau, report correlations between high CSF P-tau and higher rate of progression and cognitive decline [37]. These preliminary results do not suggest a mechanism for sTREM2 in the pathogenesis of AD, but do indicate that further studies are required to unravel the role that this protein plays in neuroinflammation and neurodegeneration.

## Conclusions

The results of our pilot study suggest that CSF sTREM2 is higher in AD subjects and may be a novel marker of glial activation in AD primarily related to neurodegenerative aspects of the disease and not Aβ pathology. The overlap between cases and controls, however, suggests that the diagnostic usefulness may be limited. Nevertheless, if replicated in larger studies, this may provide important insights into the pathogenic process in AD, and provide a potential biomarker for assessing therapeutic strategies aiming at reducing the inflammatory component of AD and thereby influencing neurodegeneration.

## Additional files

Additional file 1: Table S1. Details of patients with other neurodegenerative diseases. (DOCX 13 kb) Additional file 2: Table S2. Characteristics of AD patients and controls sampled in a replication cohort. Data expressed as mean ± SD or median (IQR) as appropriate. Probability values (p) denote differences between control and AD. A χ^2^ test was used for gender and *APOE* genotype comparisons. CSF biomarkers and sTREM2 were evaluated using the Mann-Whitney U test. (DOCX 14 kb)

## References

[1] Paloneva J, Manninen T, Christman G, Hovanes K, Mandelin J, Adolfsson R (2002). Mutations in two genes encoding different subunits of a receptor signaling complex result in an identical disease phenotype. Am J Hum Genet.

[2] Kondo T, Takahashi K, Kohara N, Takahashi Y, Hayashi S, Takahashi H (2002). Heterogeneity of presenile dementia with bone cysts (Nasu-Hakola disease): three genetic forms. Neurology.

[3] Klunemann HH, Ridha BH, Magy L, Wherrett JR, Hemelsoet DM, Keen RW (2005). The genetic causes of basal ganglia calcification, dementia, and bone cysts: DAP12 and TREM2. Neurology.

[4] Guerreiro RJ, Lohmann E, Bras JM, Gibbs JR, Rohrer JD, Gurunlian N (2013). Using exome sequencing to reveal mutations in TREM2 presenting as a frontotemporal dementia-like syndrome without bone involvement. JAMA neurology.

[5] Guerreiro R, Wojtas A, Bras J, Carrasquillo M, Rogaeva E, Majounie E (2013). TREM2 variants in Alzheimer’s disease. N Engl J Med.

[6] Benitez BA, Cruchaga C (2013). TREM2 and neurodegenerative disease. N Engl J Med.

[7] Rayaprolu S, Mullen B, Baker M, Lynch T, Finger E, Seeley WW (2013). TREM2 in neurodegeneration: evidence for association of the p.R47H variant with frontotemporal dementia and Parkinson’s disease. Mol Neurodegener.

[8] Cuyvers E, Bettens K, Philtjens S, Van Langenhove T, Gijselinck I, van der Zee J (2014). Investigating the role of rare heterozygous TREM2 variants in Alzheimer’s disease and frontotemporal dementia. Neurobiol Aging.

[9] Cady J, Koval ED, Benitez BA, Zaidman C, Jockel-Balsarotti J, Allred P (2014). TREM2 variant p.R47H as a risk factor for sporadic amyotrophic lateral sclerosis. JAMA neurology.

[10] Slattery CF, Beck JA, Harper L, Adamson G, Abdi Z, Uphill J (2014). R47H TREM2 variant increases risk of typical early-onset Alzheimer’s disease but not of prion or frontotemporal dementia. Alzheimer’s & dementia : the journal of the Alzheimer's Association.

[11] Colonna M (2003). TREMs in the immune system and beyond. Nat Rev Immunol.

[12] Neumann H, Takahashi K (2007). Essential role of the microglial triggering receptor expressed on myeloid cells-2 (TREM2) for central nervous tissue immune homeostasis. J Neuroimmunol.

[13] Paradowska-Gorycka A, Jurkowska M (2013). Structure, expression pattern and biological activity of molecular complex TREM-2/DAP12. Hum Immunol.

[14] Dubois B, Feldman HH, Jacova C, Hampel H, Molinuevo JL, Blennow K (2014). Advancing research diagnostic criteria for Alzheimer’s disease: the IWG-2 criteria. The Lancet Neurology.

[15] de Souza LC, Chupin M, Lamari F, Jardel C, Leclercq D, Colliot O (2012). CSF tau markers are correlated with hippocampal volume in Alzheimer’s disease. Neurobiol Aging.

[16] Hampel H, Burger K, Pruessner JC, Zinkowski R, DeBernardis J, Kerkman D (2005). Correlation of cerebrospinal fluid levels of tau protein phosphorylated at threonine 231 with rates of hippocampal atrophy in Alzheimer disease. Arch Neurol.

[17] Buerger K, Ewers M, Pirttila T, Zinkowski R, Alafuzoff I, Teipel SJ (2006). CSF phosphorylated tau protein correlates with neocortical neurofibrillary pathology in Alzheimer’s disease. Brain : a journal of neurology.

[18] Seppala TT, Nerg O, Koivisto AM, Rummukainen J, Puli L, Zetterberg H (2012). CSF biomarkers for Alzheimer disease correlate with cortical brain biopsy findings. Neurology.

[19] Strozyk D, Blennow K, White LR, Launer LJ (2003). CSF Abeta 42 levels correlate with amyloid-neuropathology in a population-based autopsy study. Neurology.

[20] Tapiola T, Alafuzoff I, Herukka SK, Parkkinen L, Hartikainen P, Soininen H (2009). Cerebrospinal fluid {beta}-amyloid 42 and tau proteins as biomarkers of Alzheimer-type pathologic changes in the brain. Arch Neurol.

[21] Duits FH, Teunissen CE, Bouwman FH, Visser PJ, Mattsson N, Zetterberg H (2014). The cerebrospinal fluid “Alzheimer profile”: easily said, but what does it mean?. Alzheimer's & dementia : the journal of the Alzheimer’s Association.

[22] Magdalinou NK, Paterson RW, Schott JM, Fox NC, Mummery C, Blennow K (2015). A panel of nine cerebrospinal fluid biomarkers may identify patients with atypical parkinsonian syndromes. J Neurol Neurosurg Psychiatry.

[23] Gorno-Tempini ML, Hillis AE, Weintraub S, Kertesz A, Mendez M, Cappa SF (2011). Classification of primary progressive aphasia and its variants. Neurology.

[24] Heywood W, Wang D, Madgett TE, Avent ND, Eaton S, Chitty LS (2012). The development of a peptide SRM-based tandem mass spectrometry assay for prenatal screening of Down syndrome. J Proteomics.

[25] Manwaring V, Heywood WE, Clayton R, Lachmann RH, Keutzer J, Hindmarsh P (2013). The identification of new biomarkers for identifying and monitoring kidney disease and their translation into a rapid mass spectrometry-based test: evidence of presymptomatic kidney disease in pediatric Fabry and type-I diabetic patients. J Proteome Res.

[26] Jonsson T, Stefansson H, Steinberg S, Jonsdottir I, Jonsson PV, Snaedal J (2013). Variant of TREM2 associated with the risk of Alzheimer’s disease. N Engl J Med.

[27] Kleinberger G, Yamanishi Y, Suarez-Calvet M, Czirr E, Lohmann E, Cuyvers E (2014). TREM2 mutations implicated in neurodegeneration impair cell surface transport and phagocytosis. Sci Transl Med.

[28] Matarin M, Salih DA, Yasvoina M, Cummings DM, Guelfi S, Liu W (2015). A genome-wide gene-expression analysis and database in transgenic mice during development of amyloid or tau pathology. Cell Rep.

[29] Heneka MT, Carson MJ, Khoury JE, Landreth GE, Brosseron F, Feinstein DL (2015). Neuroinflammation in Alzheimer’s disease. The Lancet Neurology.

[30] Piccio L, Buonsanti C, Cella M, Tassi I, Schmidt RE, Fenoglio C (2008). Identification of soluble TREM-2 in the cerebrospinal fluid and its association with multiple sclerosis and CNS inflammation. Brain : a journal of neurology.

[31] Craig-Schapiro R, Perrin RJ, Roe CM, Xiong C, Carter D, Cairns NJ (2010). YKL-40: a novel prognostic fluid biomarker for preclinical Alzheimer’s disease. Biol Psychiatry.

[32] Bonneh-Barkay D, Bissel SJ, Wang G, Fish KN, Nicholl GCB, Darko SW (2008). YKL-40, a Marker of Simian Immunodeficiency Virus Encephalitis, Modulates the Biological Activity of Basic Fibroblast Growth Factor. Am J Pathol.

[33] Deshmane SL, Kremlev S, Amini S, Sawaya BE (2009). Monocyte chemoattractant protein-1 (MCP-1): an overview. Journal of interferon & cytokine research : the official journal of the International Society for Interferon and Cytokine Research.

[34] Takahashi K, Rochford CD, Neumann H (2005). Clearance of apoptotic neurons without inflammation by microglial triggering receptor expressed on myeloid cells-2. J Exp Med.

[35] Lue LF, Schmitz CT, Serrano G, Sue LI, Beach TG, Walker DG (2015). TREM2 Protein Expression Changes Correlate with Alzheimer’s Disease Neurodegenerative Pathologies in Post-Mortem Temporal Cortices. Brain pathology (Zurich, Switzerland).

[36] Schott JM, Revesz T (2013). Inflammation in Alzheimer’s disease: insights from immunotherapy. Brain : a journal of neurology.

[37] Blennow K, Hampel H, Weiner M, Zetterberg H (2010). Cerebrospinal fluid and plasma biomarkers in Alzheimer disease. Nat Rev Neurol.

