# Supplementary material for: Increased cerebrospinal fluid soluble TREM2 concentration in Alzheimer’s disease
Source: Mol Neurodegener. 2016 Jan 12;11:3. doi: 10.1186/s13024-016-0071-x (PMC4709982; doi:10.1186/s13024-016-0071-x)
Supplement: Additional file 1: Table S1. — Details of patients with other neurodegenerative diseases. (DOCX 13 kb) [file 13024_2016_71_MOESM1_ESM.docx]

|  | Age at lumbar puncture | Gender | Clinical diagnosis | Aβ42 (pg/ml) | T-tau (pg/ml) | P-tau (pg/ml) | sTREM2 (pg/ml) |
| --- | --- | --- | --- | --- | --- | --- | --- |
| 1 | 56 | F | bvFTD | 471 | 177 | 20 | 114 |
| 2 | 67 | F | FTLD | 957 | 321 | 39 | 101 |
| 3 | 60 | F | Semantic dementia | 502 | 172 | - | 126 |
| 4 | 60 | F | Semantic dementia | 612 | 349 | 30 | 71.3 |
| 5 | 56 | M | CBS | 815 | 837 | 118 | 210 |
| 6 | 68 | M | DLB | 412 | 370 | 56 | 176 |

**Additional file 1: Table S1. Details of patients with other neurodegenerative diseases.**
