# Supplementary material for: Increased cerebrospinal fluid soluble TREM2 concentration in Alzheimer’s disease
Source: Mol Neurodegener. 2016 Jan 12;11:3. doi: 10.1186/s13024-016-0071-x (PMC4709982; doi:10.1186/s13024-016-0071-x)
Supplement: Additional file 2: Table S2. — Characteristics of AD patients and controls sampled in a replication cohort. Data expressed as mean ± SD or median (IQR) as appropriate. Probability values (p) denote differences between control and AD. A χ2 test was used for gender and APOE genotype comparisons. CSF biomarkers and sTREM2 were evaluated using the Mann-Whitney U test. (DOCX 14 kb) [file 13024_2016_71_MOESM2_ESM.docx]

| Subject details | Controls (n=16) | AD patients (n=24) | P value |
| --- | --- | --- | --- |
|  |  |  |  |
| Gender (F/M), no (%) | 9(56)/7(44) | 13(54)/11(46) | 0.9 |
| Age (years), mean ± SD | 55.6 ± 9.7 | 64.3 ± 6.8 | 0.0071 |
| MMSE Score, median (IQR) | 30.0 (29.0 – 30.0) | 21.0 (14.0 – 25.0) | < 0.0001 |
| *APOE* ε4 positive (%) | 31% | 69% | 0.1053 |
| CSF Biomarkers |  |  |  |
| Aβ1-42 (pg/ml), median (IQR) | 1013.0 (883.8 – 1211.0) | 357.0 (259.0 – 460.3) | <0.0001 |
| T-tau (pg/ml), median (IQR) | 276.5 (196.3 – 332.5) | 777.0 (430.3 – 1029.0) | <0.0001 |
| P-tau (pg/ml), median (IQR) | 32.0 (24.8 -40.3) | 80.0 (56.5 – 97.0) | <0.0001 |
| T-tau/Aβ1-42 ratio, median (IQR) | 0.3 (0.2 – 0.3) | 2.1 (1.2 – 3.2) | <0.0001 |
| sTREM2 (pg/ml), median (IQR) | 171.3 (153.5 – 241.5) | 230.5 (166.5 – 297.4) | 0.0312 |

**Additional file 2: Table S2. Characteristics of AD patients and controls sampled in a replication cohort**.

Data expressed as mean ± SD or median (IQR) as appropriate. Probability values (p) denote differences between control and AD. A χ^2^ test was used for gender and *APOE* genotype comparisons. CSF biomarkers and sTREM2 were evaluated using the Mann-Whitney U test.
